# Pathology Update for Urologists

**DOI:** 10.1155/2011/183761

**Published:** 2011-09-25

**Authors:** Kenneth A. Iczkowski, Rodolfo Montironi, Dengfeng Cao, Cristina Magi-Galluzzi, M. Scott Lucia

**Affiliations:** ^1^Urology Specialists of America, 2100 Marble Cliff Office Park, Suite A, Columbus, OH 43215-1056, USA; ^2^Institute of Pathological Anatomy and Histopathology, School of Medicine, Polytechnic University of the Marche Region (Ancona), United Hospitals, Via Conca, 71, Ancona, 60126 Torrette, Italy; ^3^Arizona Cancer Center, College of Medicine, The University of Arizona, 1515 North Campbell Avenue, Tucson, AZ 85724-5024, USA; ^4^Department of Pathology and Immunology, Washington University School of Medicine, 660 S Euclid Avenue, Campus Box 8118, Saint Louis, MO 63110, USA; ^5^Department of Anatomic Pathology and Glickman Urological and Kidney Institute, Cleveland Clinic, Lerner College of Medicine, 9500 Euclid Avenue, OH 44195, USA; ^6^Department of Pathology, University of Colorado Health Science Center, Aurora, CO 80045, USA

This special issue of *Prostate Cancer* was designed to be read by both urologists and pathologists. It comprises eight papers, of which 3 are original studies and 5 are review papers, largely devoted to promoting urologists' understanding of the histologic findings provided by pathologists in both biopsy and radical prostatectomy specimens.

Many of the papers are closely related and fall into 4 general subheadings: (1) technical implications of biopsy sampling and submission; (2) correlation of histoarchitectural findings with tumor development and outcome; (3) prognostic factors; (4) tumor biology.

*Biopsy sampling and submission technique* is addressed in one original article and one review article. D. Parada et al. emphasize the cost savings attainable by inking needle cores from various sites submitted in the same block. Galosi et al. review their work and that of others on the benefits of inking the peripheral end of each core for orientation and stage prediction. 

*Correlation of histoarchitectural findings with tumor development* is explored in an original study and a review. K. A. Iczkowski et al. based their study on the observation that not all prostate cancers graded as Gleason 4 look alike. Thus, a quantitative analysis of the density of cancer epithelial cells compared with stroma and the loss of ability to form a lumen (spaces in cancer are termed pseudolumens) may give us additional, cost-effective information to stratify outcomes more finely. The review by R. Nagle and B. Cress ties in with the Iczkowski study, by postulating that loss of polarity and the ability to form lumens are key measures of tumor aggressiveness. They review the literature and postulate that tubule formation, rather than epithelial-mesenchymal transition (EMT) is the main driver of invasion and metastasis. 

*Prognostic factors* are covered in three review articles. Kotb et al. provide an overview of prognostic factors with respect to recurrence. Iczkowski and Lucia review prostatectomy margin status, a key predictor of outcome whose implications may not be well understood by urologists. The location, extent, and grade of margin positivity, it turns out, can modify its importance. P. Furtado et al. review the outcome of a particularly aggressive variant of prostate cancer called small cell carcinoma. This constitutes fewer than 1% of prostate cancers and, importantly, tends not to elevate serum PSA.

Finally, the *tumor biology* of prostate cancer is studied by P. N. Werahera et al. using molecular techniques to estimate tumor doubling time. They postulate a role for apoptosis in determining tumor aggressiveness.

We are pleased to present this compendium of papers containing the most contemporary thought regarding the submission of specimens, new insights on histologic morphology, prognostic implications of findings, and tumor biology. We hope we have stimulated urologists to understand and take interest in these up-to-the-minute developments in pathology that affect their practice.

*Kenneth A. Iczkowski* *Kenneth A. Iczkowski* *R. Montironi* *R. Montironi* *Dengfeng Cao* *Dengfeng Cao* *Cristina Magi-Galluzzi* *Cristina Magi-Galluzzi* *M. Scott Lucia* *M. Scott Lucia*

